# Short Peptides as Powerful Arsenal for Smart Fighting Cancer

**DOI:** 10.3390/cancers16193254

**Published:** 2024-09-24

**Authors:** Joanna Bojarska, Wojciech M. Wolf

**Affiliations:** Chemistry Department, Institute of Inorganic and Ecological Chemistry, Łódź University of Technology, S. Żeromskiego Str. 116, 90-924 Łódź, Poland; wojciech.wolf@p.lodz.pl

**Keywords:** short peptides, self-assembled peptides, cancer, therapy, diagnosis, vaccine, antigen, adjuvant, vaccine delivery, immunotherapeutic drugs

## Abstract

**Simple Summary:**

This mini-review summarizes the up-to-date progress in developing anticancer therapies based on cutting-edge strategies employing short peptides. This work serves as a guide to the versatility of short peptide-based applications in cancer diagnosis and treatment. Starting with a brief introduction to the unique features of short peptides and peptide self-assemblies, including their pros and cons, we outline short peptide sequences in terms of their specific functions, such as immunotherapeutic drugs, supramolecular vaccines, antigens, and adjuvants. We highlight self-assembled peptides as scaffolds for targeted delivery or co-delivery of multiple therapeutics and genes. We also emphasize the relevance of short peptides in combinatorial immuno- and traditional therapies, 3D culture, and cancer imaging. Finally, we explore ultra-short natural bioactive peptides as potential anticancer agents.

**Abstract:**

Short peptides have been coming around as a strong weapon in the fight against cancer on all fronts—in immuno-, chemo-, and radiotherapy, and also in combinatorial approaches. Moreover, short peptides have relevance in cancer imaging or 3D culture. Thanks to the natural ‘smart’ nature of short peptides, their unique structural features, as well as recent progress in biotechnological and bioinformatics development, short peptides are playing an enormous role in evolving cutting-edge strategies. Self-assembling short peptides may create excellent structures to stimulate cytotoxic immune responses, which is essential for cancer immunotherapy. Short peptides can help establish versatile strategies with high biosafety and effectiveness. Supramolecular short peptide-based cancer vaccines entered clinical trials. Peptide assemblies can be platforms for the delivery of antigens, adjuvants, immune cells, and/or drugs. Short peptides have been unappreciated, especially in the vaccine aspect. Meanwhile, they still hide the undiscovered unlimited potential. Here, we provide a timely update on this highly active and fast-evolving field.

## 1. Introduction

Cancer is still one of the world’s largest health problems and one of the leading life-threatening diseases around the world [1]. It is estimated that nowadays there are ~20 million new cases and ~10 million cancer-related deaths annually. By 2040, the number of new cases can rise to ~30 million, while the number of deaths can rise to more than 15 million [1]. Cancer therapies have taken the front seat in biomedicine. Despite the progress in cancer treatment in the last decades, the determination of versatile strategies with either high biological safety or effective modulation of immune responses is still challenging [1,2]. In 2022, Hanahan proposed several key hallmarks for cancer initiation and progression related to the most recent studies [2]. These concepts allow us to reevaluate existing drugs, providing a more ‘foresighted’ perspective for future development of anticancer therapeutics. Cancer is related to different diseases maneuvered at once rather than a singular one; hence, treatment should be rather multidirectional. We should remember that cancer is an ‘intelligent disease’—it escapes from the control of the immune system by deceiving the immune mechanisms and becomes unrecognizable to the immune system. Therefore, we need intelligent therapeutic strategies.

Peptides are ‘smart by nature’. Notably, peptides, chains of amino acids, constitute proteins in organisms and play crucial roles in life processes. They can provide specific recognition related to biomolecules and can mimic the protein features, which conventional chemical molecules cannot do. Thus, peptides have unique natural possibilities to associate with cell components [3,4]. They are well suited to this role because peptides combine features of large biological entities and chemical small molecules, occupying an intermediate position between them [3,4]. Peptides are the most versatile biomolecules. They offer a huge spectrum of functional and structural diversity. On the other hand, the clinical applications of peptide therapeutics are limited due to peptide limitations such as enzymatic degradation and weak oral bioavailability [3,4]. Nevertheless, diverse peptide modifications (cyclization, use of D- and non-natural amino acids, etc.), modern bio-informatics as well as advanced bio-chemical methodologies, and recent great biotechnological progress help to overcome these obstacles [3,4]. At least two main chemical techniques for peptide production can be mentioned, such as solid-phase and solution-phase peptide synthesis, both of which are economically suitable for shorter peptides. In the context of biological methods, enzymatic synthesis, isolation of bioactive peptides from natural sources by extraction, semisynthesis, or recombinant DNA technology, as well as other technologies (i.e., mirror-image phage display technology) are worth mentioning. In our global review, we present a SWOT analysis of peptides in detail as well as a comprehensive description of short peptide synthesis [4].

Short peptides have additional unique features: they are easy to synthesize, exhibit greater conformational stability, and can penetrate solid tumor tissues [4]. They also demonstrate remarkable selectivity, especially for drug targets on the cell surface. Additionally, from the drug point of view, the structure of small peptides may be perfectly tuned to prevent allergies and autoimmune reactions caused by contaminants [3].

Peptide-based structures undergoing self-assembly have enhanced stability and a simpler synthesis process with reduced costs [5]. It is worth noting that the self-assembly is spontaneous generation of ordered structures in the nanoscale, commonly observed in nature [5]. Complex bio-structures, cell membranes, organelles, cellular cytoskeleton, extracellular matrix, or collagen in the extracellular matrix are good examples [5]. Cell-membrane integrins, in turn, are related to molecule recognition between the cells and the extracellular matrix itself [5]. Molecular self-assembly gives a novel opportunity for the formation of different highly ordered nanostructures, driven by thermodynamics and governed by kinetics, which are useful in cancer therapies [6,7]. Peptides can self-assemble into different structures, including inter alia nanospheres, nanofibers, or nanocapsules. Peptide structure is correlated with its self-assembly behavior through non-covalent interactions, i.e., hydrogen bonding. Modulation of these interactions promotes nanomedicines with controllable pharmacokinetics [6,7]. Self-assembled peptide-based nanomaterials may deliver therapeutic or imaging agents (or a combination of both) to safeguard the drug in the blood (by their structural conformations) or maintain the integrity of the drug before it reaches the acquired target to get the full effect. Consequently, this leads to better effectiveness and decreased accumulation of drugs in body organs [6,7]. Self-assembling of shortened sequences of peptides leads to supramolecular particles that are very small and ideal for making vaccines [8]. Self-assembled peptides sensitive to the cellular environment surrounding the tumor, especially in terms of the biomarkers, are perfect platforms for effective cancer imaging [9,10].

Overall, peptide assembles, with their natural components, excellent cytocompatibility, and controllable morphologies, have been broadly employed in the combinatory conventional theranostic (therapeutics and diagnostics) and immunotherapy approaches [8,9,11,12].

Despite the huge potential of short peptides in modern safe and effective therapies and diagnosis, their importance in the progress of cutting-edge anticancer strategies has not been thoroughly summarized as yet.

This review presents recent progress in cancer treatment and imaging in terms of the advantages of short peptides and their diverse functions in therapeutic processes and imaging in the fight against cancers. We highlight short peptide-based immune therapeutics and delivery scaffolds. We outline combinatorial approaches involving immunotherapy, chemo- and radiotherapy, as well as photodynamic therapy.

## 2. Short Peptide Immunotherapeutic Drugs

Immunotherapeutic drugs are becoming increasingly important due to their specific action inhibiting protein-protein interactions via multiple cell types [13]. Peptide-based therapeutics combine the advantages of monoclonal antibody drugs and small chemical agents. They have exceptional target selectivity on the cell surface and excellent penetrability in solid tumors [14].

### 2.1. Peptide-Based Immune Checkpoint Blockades

Immune checkpoints are the negative regulators of the immune system. They keep homeostasis and protect autoimmunity against attacking cells uncritically [9,15]. Nonetheless, immune checkpoints may turn on in tumor cells to block the appearing anticancer response, resulting in tumor cell growth [16,17]. Blockers of these checkpoints cause inhibition of the immune escape of tumor cells, activating the immune cells (e.g., T cells) to secure from tumor cells. It is a full-of-promise approach used in clinical trials [18].

The main inhibitory receptors represented by activated T cells contain PD-1, T-cell immunoglobulin 3, T-cell immunoglobulin, lymphocyte-activation gene 3, CTLA-4, and ITIM domain.

PD-1 immune checkpoint is a member of the extended B7/CD28 family and is greatly correlated with activated T (and B) cells, dendritic cells, natural killer cells, and tumor-related macrophages. PD-1 is related to mature T cells and controls effector T cell action in the environment of the tumor. The ligands for PD-1 containing PD-L1 and PD-L2 are manifested via antigen-presenting cells and cancer cells. Binding PD-1 with its ligands deactivates T-cell kinase, thereby decreasing the generation of inflammatory cytokines and optimizing T-cell activity. Thus, blockades for the PD-1/PD-L1 pathway provide the action of tumor-specific T cells to re-recognition of the immune system and storm cancer cells; hence, protecting from the immune escape of cancer cells. PD-1 and PD-L1 are inspirations for the rational design of biomimetic short peptides as inhibitors [9]. Peptides are appealing alternatives to monoclonal antibodies, overcoming their limitations such as low ingress via solid-tumor tissues, poor stability, insufficient strategies of administration, hard-to-control pharmacokinetic profile, restricted ability to stimulate cytotoxic immune responses via macrophages and natural killer cells, and large production costs [9]. Short peptides, mainly due to their great potency in modulating the immune system, extraordinary biocompatibility, and easy synthesis, have attracted broad attention [14].

It should be mentioned that the peptides containing L-amino acids have poor stability related to enzymatic degradation. Therefore, diverse structural modifications and modern methodological strategies are applied. BMS-986189 [(Met)-Tyr-Ala-Asn-Pro-(Dpr)-Leu-(Hyp)-Trp-(Dab)-Trp-(Nle)-(Nle)-Leu-Cys-Gly], targeting PD-L1, is an example of macrocyclic peptide undergoing a phase I clinical trial [14,19]. OPBP-1 (GQSEHHMRVYSF) peptide loaded with trimethyl chitosan hydrogel, on the other hand, is the first example of a peptide for oral administration for immune checkpoint blockade, obtained by mirror-image phage display technology. It presents significantly increased oral bioavailability and half-life in mouse models [20]. In addition, DTBP-3 (GGYTHWHRLNP) is the first D peptide blocker of the TIGIT/PVR (poliovirus receptor) interaction [21].

Furthermore, we should mention short peptide inhibitors that target immune checkpoints in a selective way on other immune cell subcategories.

The fascinating issue of ‘cold tumors’, differentiated through a lack of T-cell infiltration in specific cancer tissues, is a major setback in immunotherapy. However, the majority of solid tumors have a significant number of immune cells called macrophages, which play key roles in the tumor microenvironment. Notably, some immune checkpoints such as CD47/SIRPα and CD24/Siglec10 successfully hamper macrophage phagocytosis, thus disrupting the removal of cancer cells via macrophages [20]. Pep-20 and its analog pep-20-D12 containing terminal D-amino acids (awsATWSNYwrh) can be such a potential inhibitor [14,20]. More examples of short peptides serving as immune checkpoint inhibitors are summarized in Table 1.

### 2.2. Peptide-Drug Conjugates and Dual-Function Short Peptides

Peptide-based drug conjugates containing non-toxic therapeutic agents related to immunotherapeutic targets and peptides that target non-endocytic receptors are future-proof due to their unique mechanisms of action preventing unpredicted toxicity in terms of toxins in antibody-drug conjugates [14].

Notably, peptide-drug conjugates, contrary to antibody-drug conjugates, may be much more easily linked to small chemical drugs. It simplifies synthesis. Here, we should refer to amphiphilic peptide drug conjugates that are developed via conjugation of the small radiosensitizers with the PD-1/PD-L1 blocking peptide DPPA-1. The self-assembly of these conjugates leads to the generation of nanoparticles that effectually encapsulate TLR7/8 agonists. This complex process has noticeable synergistic effects. It means that sensitization of radiotherapy, innate immunity activation, and strengthening adaptive immune responses are realized together [32].

In addition, it is worth mentioning one more example—the DPPA-1 peptide conjugated with the drug doxorubicin via utilizing a substrate sequence developed for matrix metalloproteinase 2 (MMP2). It resulted in the formation of nanoparticles sensitive to the enzymatic action of MMP2. These nanoparticles have a dual function—activating either chemotherapeutic cytotoxicity or T-cell-mediated immune killing actions in an impressive way [33].

Dual-function peptides like bispecific antibodies are peptide-based moieties conjugated to functionally target two complementary moieties, allowing for concurrent modulation of separate signaling pathways; hence, displaying multifaceted modulatory outcomes. Dual-function peptides are appealing alternatives for bispecific antibodies because they are much easier to design and synthesize [14]. Pal-DMPOP (Pal-PEG4-WSMTWWNYWrvysf), designed by linking the basal active part of the PD-1/PD-L1 blocking peptide OPBP-1 with the fundamental part of the CD47/SIRPα peptide inhibitor pep-20, can be a good example of this kind of peptide. It shows immunomodulatory action via selective involvement and modulation of the signaling pathways related to either T-cell or macrophage checkpoints [34]. It resulted in the activation and improvement of anticancer immune responses [34]. Another example can be ^D^SPOGS, a long-acting peptide, engineered by the conjugation of OPBP-1 with the anti-angiogenesis peptide ^D^A7R, that demonstrates high anti-tumor activity by disrupting neovascularization in cancer tissues, and next to the albumin-binding peptide ^D^SP [14,35].

Furthermore, we can mention a glycopeptide-based in vivo self-assembled bispecific nanoantibody, LGASWHRPDKK(PLGYLG-(man)3-LVFFAECG, using the MMP-2 enzyme. It targeted the CD206 receptor of M2 tumor-associated macrophages and the C-X-C chemokine receptor 4 of tumor cells at the same time. LGASWHRPDKK(PLGYLG-(man)3-LVFFAECG repolarized M2 in M1 tumor-associated macrophages and engaged cytotoxic T cells. Simultaneously, LGASWHRPDKK(PLGYLG-(man)3-LVFFAECG blocked C-X-C chemokine receptor 4 downstream signaling in the long term. Thus, it decreased the metastasis of the tumor and supported T-cell infiltration. This supramolecular peptide-based therapeutic agent is a new strategy to develop cutting-edge bispecific antibodies [36].

## 3. Peptide-Based Cancer Vaccination

Vaccines are extremely needed for public health intervention in oncology [37,38]. Cancer vaccines have been under intense studies for 40 years. In 1891, William Coley ‘injected live and heat-killed bacteria into the bone and soft tissue sarcomas’. It was the first successful trial to control the immune system in the context of cancer [39]. Despite many years of research on cancer vaccines, it has not been possible to achieve meaningful clinical success due to low antigens stability, immune risks related to contaminants, the off-targeting-induced unwanted autoimmunity, and problems with production and transport to lymph nodes, in terms of traditional vaccination therapy [9,40]. Nevertheless, the latest progress in the identification and formulation of antigens as well as procedures of combination therapies are promising [41].

Overall, cancer vaccines instruct the immune system to recognize cancer antigens as foreign [42]. These vaccines can be used prophylactically to prevent cancer disease or therapeutically to fight the disorder [43].

There are two types of prophylactic vaccines against cancers approved by the Food and Drug Administration (FDA). They are related to cancer-causing viruses such as human papillomavirus (HPV; vaccines called Gardasil and Cervarix, formed from viral-like particles containing the single capsid protein L1) and hepatitis B virus (HBV; vaccines called Engerix-B, Recombivax HB, and Heplisav-B, that ware viral-like particles utilizing hepatitis B surface antigens produced in yeast). The effectiveness of these vaccines is related to the generation of a strong neutralizing antibody response in terms of immunodominant viral antigens [43].

Therapeutic cancer vaccines are not the same as prophylactic vaccines. They cause an immune response to tumor cells that have endured previous therapies [44]. In the context of cancer type, tumors can be removed during surgery, but not completely. Immunotherapy includes key stages of immune responses towards an antigen. In particular, stimulation of both cancer-specific effector and cytotoxic tumor-directed T cells, next recognition, and finally tumor killing [44].

Three therapeutic cancer vaccines have been fully FDA-approved so far. There are as follows: Bacillus Calmette-Guerin (called BCG; from Mycobacterium bovis, for nonmuscle invasive bladder cancer), Sipuleucel-T (Provenge; for prostate cancer; by using the immune cells such as dendritic cells, B and T cells, and natural killer cells isolated via leukapheresis to stimulate an immune response toward prostatic acid phosphatase), and talimogene laherparepvec (T-VEC; a genetically modified oncolytic virotherapy extracted from herpes simplex virus type I, patients with melanoma following initial surgery) [45].

Nevertheless, a total of seven cancer vaccines have been approved for commercial use outside the United States, but largely without Phase 3 trial data: Hybricell (Brazil, 2005), Creavax-RCC (Korea, 2007) (renal carcinoma), Oncophage (Russia, 2008; protein-peptide vaccine; but withdrawn due to concerns over data integrity), M-vax and DCvax-Brain (melanoma and glioblastoma, Switzerland, 2014), CIMAVAX-EGF (lung cancer; Cuba, 2016), APCEDEN (lung, colorectal, ovarian, prostate cancers) (India, 2017). CIMAVAX-EGF is now undergoing early-stage, US-based clinical trials (NCT04298606, NCT02955290) [41].

### 3.1. Peptide-Based Cancer Vaccines

Over the years, the progress of the development of cancer vaccines has been modified ‘from entire, inactive, or attenuated viruses to subunit components’ [46].

Peptide vaccines consist of short protein fragments, known as peptides, constituted from popular or predicted cancer antigen epitopes [46].

The length of the sequence in peptide vaccines has relevance in the advancement of a robust immunogenic response. Overall, short peptide vaccines can have 8–10 amino acid residues, while longer analogs—11–30 amino acids in the context of major histocompatibility complex (MHC) class I or II molecules, respectively. Short peptides express the nominal epitope able to bind the class I MHC moieties. Short peptides are attractive for vaccination due to their specific features, such as easy synthesis and cheap production on a clinical scale. Nevertheless, they can bind to the MHC of non-professional antigen-presenting cells (together with B and T lymphocytes) that do not have the secondary signaling machinery and do not give the full range of costimulatory signals needed to complete T-cell activation [47]. In addition, this non-conventional antigen presentation occurs in non-inflamed lymph nodes without a strong pro-inflammatory context [48]. It could result in poor T-cell response or immune tolerance [48]. Small peptides are ‘strictly HLA-type restricted’; thus, they must match the patient’s HLA in a perfect way, which restricts their utilization to a specific subset of patients [49]. Moreover, short peptides are prone to fast exopeptidase-mediated degradation, and consequently, they have a limited half-life. On the other hand, they are exactly the natural HLA ligands on cancer cells, providing the opportunity for precise immuno-monitoring characteristics [50].

The advantages of peptide cancer vaccines are as follows: simple chemical-based synthesis, straightforward and expedient manufacturing, cost-effectiveness, flexibility to multiple antigens, high specificity and stability, safety for clinical application, inducing both humoral and adaptive immunity systems, immunodominance, minimal side effects, and potential for personalization [51,52,53].

The disadvantages of peptide cancer vaccines are as follows: relatively poor immunogenicity, tumor heterogeneity, inappropriate adjuvants, antigen loss, low MHC expression, lack of T-cell infiltration in the tumor tissue, and inducing immune suppression through T-cell dysfunction [43,51,54].

Nevertheless, there are different possibilities to decrease the limitations of peptide vaccination. In particular, in the context of low immunogenicity, peptide-based vaccines are usually formulated with immune adjuvants. Overall, synthetic toll-like receptor (TLR) ligands and peptides such as PADRE (aKXVAAWTLKAa, where X is cyclohexyl-alanine and a is D-alanine) are used to boost the immune responses [55]. To increase the immunogenicity of peptide antigens, heteroclitic peptides are helpful to enhance binding affinity to the MHC moieties. Heteroclitic peptides are modified peptides that have been altered via replacing amino acids in the epitope with similar biochemical properties, structure, and function. They may stimulate an enhanced immune response towards specific antigens [56,57].

In terms of the short half-life and poor stability of peptides in the body, antigen peptides are included in delivery systems [58].

Overall, some peptide vaccine strategies have been analyzed in diverse types of tumors in recent years, but imperfect effects have been reported [59]. Tecemotide, ISCOMATRIX, DepoVax, Lipo-MER [60], or dendritic cell-derived exosomes (DEXs) [61,62], can be good examples. However, DEXs still hold promise as a part of combination therapies [63].

Only one peptide cancer vaccine, namely Provenge^®^ (Sipucleucel-T) against castration-resistant prostate cancer, has been registered so far. In addition, other peptide cancer vaccines are developed for prostate cancer, inter alia cancer-associated membrane carbohydrates such as mucin 1 (MUC1), ganglioside (GM2), Thompson-Friedenreich antigen, and globo H. More specifically, GVAX, DCVAC/PCa, a multi-epitope peptide vaccine, sipuleucel-T, and PROSTVAC strategies have been investigated in phase III trials. More specifically, they demonstrated good safety and immunological effects; however, no impressive clinical effectiveness [63].

On the other hand, numerous peptide vaccine formulations are under (pre)clinical studies (Table S1). Notably, peptide cancer vaccines are employed in ~35% of ongoing clinical trials, mainly in Phase 1 or ½. They are mainly used to treat brain (15%), lung (12%), breast (10%), and skin (8%) cancers. Most of these vaccines target tumor-associated antigens (with hTERT and HER2 as most commonly targeted). Neoantigen targets are related to ~30% of studied vaccines. The poly-ICLC and Montanide are the most common adjuvants. These vaccines are mainly administered via subcutaneous and intradermal routes. Interestingly, only ~20% of trials concern monotherapies [41]. Peptide cancer vaccines are connected with immune checkpoint blockades, chemotherapy, and cytokine therapy [41].

### 3.2. Peptide-Based Neoantigen Cancer Vaccines

Cancer vaccines related to neoantigens are beginning to receive extensive attention due to their specific and durable T-cell immune response with nonspecific toxicity. The concept of this kind of vaccine was proposed in the 1990s, but there were no high-throughput sequencing technologies and bioinformatics tools until now [64]. Neoantigens, called TSAs (tumor-specific antigens), are unique peptides obtained from non-synonymous somatic mutations in cancer cells. More specifically, neoantigens can arise from these specific mutations in the coding area of endogenous translocations, splice variants, and integrated retrotransposons or bacteria [65]. Notably, they are absent in normal cells.

The recognition of neo-antigens specific to individual patients is achievable due to the possibility of next-generation sequencing [55].

Personalized neoantigen vaccines could be able to evoke long-lasting, robust immune responses, resulting in effective tumor elimination and preventing recurrence. Clinical trials revealed exciting outcomes as either monotherapy or combined therapy with checkpoint inhibitors [66].

Generally speaking, the platforms of personalized neoantigen vaccines are represented by peptide vaccines, nucleic acid vaccines, and neoantigen-pulsed dendritic cell (DC) vaccines [67,68]. Among them, neoantigen-based peptides have unique features such as exact sequence, stable chemical properties, and low toxicity. They are easy to synthesize at low costs.

Peptide-based neoantigen vaccines are the most common platform among all types of neoantigen vaccines in either clinical trials or early-stage studies. Overall, it can be mentioned that vaccines based on the longer peptides must be endocytosed, processed through activated antigen-presenting cells, and then bound to MHC molecules. The binding of longer sequences to MHC II molecules turns on CD4^+^ T cells [69].

NeoVax is a vaccine of up to 20 predicted individual tumor neoantigens inducing multifunctional CD4^+^ and CD8^+^ T cells to target 60% and 16% of new neoantigens observed in patients with cancers, respectively. In a follow-up of patients with melanoma, the cancer did not return in most patients, while the others achieved tumor regression when combined with anti-PD-1 [70].

Thus, the neoantigen peptide vaccines activate neoantigen-specific T cells in vivo as well as ensure that the combined treatment improves the therapeutic effect. In addition, these vaccines are appealing in the fight against nonsolid tumors such as glioma or leukemia [71,72].

Neoantigen peptide-pulsed dendritic cells (DC) vaccines have appealing efficacy in the treatment of some malignant tumors due to their high immunogenicity, safety, specificity, and promising long-lasting immunity [66]. They present an extended range and heterogeneity of neoantigen-specific T cells in either melanoma cancers or advanced lung cancer [73,74]. Some of the clinical studies are worth a broader description. Neoantigen short peptide-loaded DC vaccines stimulate a T-cell specific immune response, leading to antigen escalating in patients with melanoma [75]. The Neo-DCVac vaccine showed significant therapeutic potency in patients with lung cancer [73]. The Neo-MoDC vaccine utilized in combination with ICI therapy is another case that revealed total regression in metastatic gastric cancer [76].

To sum up, neoantigen peptide vaccines, contrary to other vaccines, can induce a greatly targeted immune response, better safety and stability, limited antigenic complexity, simplified manufacturing process, and can carry multiple epitopes [51]. Nevertheless, peptide vaccines are prone to the obstacles of immunological adjuvants, and neoantigen identification. They are weakly immunogenic in the immunosuppressive microenvironment of tumors. In consequence, neoantigen peptide vaccines face the problem of either adjuvant screening or combination approaches in cancer therapy [66,77].

### 3.3. Antigens and Adjuvants

Short peptides with small antigenic epitopes to bind with receptors have gained importance in recent years as appealing vaccinations. The precise structure and easy production of short peptides are key reasons [78,79]. It can be mentioned that suitable antigen affinity for the Toll-like receptor (TLR)/MHC receptors is essential to stimulate the generation of CD4^+^ or CD8^+^ T cells for immune responses. Peptide antigens have reduced immunogenicity; hence, peptide adjuvants are employed to support the activation of their immune response [80]. The design of short peptide-based antigens to stimulate significant and long-term immunity towards viruses was inspired by the subunits of the native proteins in terms of the revealed protein sequences in organisms or virus vaccines (Table 2). Overall, antigens associated with MHC class I receptors related to activation of CD8^+^ T cells contain 8–10 amino acids, while the antigens associated with MHC class II receptors and CD4^+^ T cells have 13–18 amino acids [78].

Peptide vaccines are popular in inter alia immunotherapy of melanoma [81]. In this context, the majority of antigens originate from ‘melanocyte differentiation proteins’ called tyrosinases, especially MART-1 (Melan-A), and glycoprotein 100 (gp100) [82]. They primarily stimulate the formation of cytotoxic T lymphocytes (CTLs) for immunity. Tyrosinase, the rate-limiting enzyme in the synthesis of melanin, consists of two immunogenic peptides such as tyrosinase_1–9_ and tyrosinase_368–376_ [83]. The latter domain in which asparagine at position 3 was replaced by aspartic acid had amazing immunogenicity. In addition, the MART-1_26–35_ domain was utilized as antigens in clinical trials [84,85]. In terms of protein gp100, the domain of gp100_280–288_, exhibited through either melanoma or healthy melanocytes, led to immunogenicity, even with low production of CTL [86]. The enhancement of the immune response was achieved by a linking vaccine-restricted domain gp100_209–217_ with an immune activator interleukin-2 (IL-2). Alternative melanoma antigens are a hydrophilic epitope HGP100_25–33_ and a hydrophobic epitope in tyrosine-based protein 2 (TRP2_180–188_).

A nanovaccine via the integration of nanoparticles composed of poly(D,L-lactide-co-glycolide) functionalized with antigen HGP100 and adjuvant monophosphoryl lipid with liposomes coated with mannose was formulated by Guo and colleagues [87]. For the progress of vaccination, the antigen HGP100 peptide was linked to the ‘immune-stimulatory spherical nucleic acid’ by a group of Mirkin [88].

Wakabayashi and coworkers employed a solid-in-oil nanodispersion in the role of nanocarriers in the antigen co-delivery based on modified TRP-2 peptide (KKKGSVYDFFVWL) and adjuvant resiquimod (R-848) [89]. This conjugate showed either the significant inhibition of melanoma growth or suppression of lung metastasis. Moreover, antigens HGP100 and TRP2 were co-encapsulated into empty mesoporous silica nanoparticles. It was efficacious in the stimulation and maturation of dendritic cells (DC) as well as secretion of tumor necrosis factor-α (TNF-α), IFN-γ, IL-12, and IL-4 to stimulate immunity [90].

Peptide antigens are epitopes extracted from ovalbumin, such as OVA_250–264_, OVA_253–266_, OVA_257–264_, and OVA_323–339_. They stimulate CD8^+^ cytotoxic T-cell immune responses. In the context of using the nanocapsules consisting of 60 nonviral E2 subunits of pyruvate dehydrogenase, Wang et al. reported a viral-mimicking multifunctional vaccine platform by encapsulation of antigen OVA_257–264_ and oligonucleotide adjuvant cytosine-guanine motif (CpG) [91]. It demonstrated complementary spatiotemporal transport of therapeutics to DCs and improved generation of CD8^+^ T cells as well as immune activation. Additionally, extending the OVA_257–264_ epitope to CCYSIINFEKL with two thiol groups made possible in situ formation of fluorescent antigen-gold nanoclusters (peptide-AuNCs) with corrected immunostimulatory [92]. In addition, the immunity was upgraded via co-loading CpG adjuvant on the surface of AUNC. What is more, Zhang et al. developed an ultra-short ‘nanovaccine functionalized with scavenger receptor class B1 targeting mature dendritic cells’ that is biocompatible. It delivered peptide antigens such as OVA_257–264_, OVA_323–339_, and HGP_10025–33_ to lymph nodes in a productive manner [93].

Furthermore, we can mention membrane-binding glycoprotein mucin 1 (MUC1), which is essential in the protection of epithelial surfaces and signaling transduction. Overall, it is upregulated with glycosylation mutation in different cancer types, including breast cancer, myelomas, lymphomas, or pancreas cancers; hence, MUC1 becomes immunogenic [94]. This issue was the catalyst for the development of antigens useful for stimulation of MUC1-associated cytotoxic T lymphocyte response. MUC1 is transmembrane glycoprotein type I that is accented by an extracellular domain containing changeable sequences (PDTRPAPGSTAPPAHGVTSA) and an upper level of glycosylation on serine and threonine in all repeats of tandem. Therefore, MUC1-based peptide antigens can be developed in terms of MUC1 epitopes. Vaccines reported by Huang and colleagues through the conjugation of the HGVTSAPDTRPAPGSTAPPA sequence with glycosylated threonines (at 9 or 16 positions) to an assembling domain Q11 can be a good example [95]. These vaccines based on B cell epitopes caused remarkable cytotoxic T-cell immune responses stimulated through type I T-helper cells. Moreover, MUC1-based vaccines related to the covalent binding antigen candidate glycopeptide tandem repeat TSAPDTRPAP with an assembling sequence Nap-G^D^F^D^F^D^Y^D^K were engineered by Zhao et al. [96].

Moving forward, other immunogenic proteins are used to design peptide vaccines. An epitope E75 extracted from HER_2_/neu (a proto-oncogene in numerous epithelial cancers) developed for the treatment of breast cancer can be one of the examples [97].

In addition, a WT1 Pep427 derived from Wilm’s tumor protein (WT1) is another immunogenic antigen that was conjugated with single-wall carbon nanotubes to elicit a fast specific IgG response [98].

New York esophageal squamous cell carcinoma-1′ (NY-ESO-1) is an immunogenic tumor testis antigen strongly manifested in diverse cancers such as melanoma and breast cancer. It also induces T-cell-associated immunity [99].

Gazzinelli et al. reported another vaccine scaffold by linking antigen NY-ESO-1 and adjuvant CpG DNA to carbon nanotubes (CNT) [100].

Wang et al. utilized E2 viral-like capsules for both encapsulating antigens NY-ESO-1 and HLA-A2 to correct the low immunogenicity of individual antigens [101].

Moreover, the epitopes extracted from either human papillomavirus (HPV) such as HPV16 E7_11–20_ [102], E7_43–57_ [11,103,104], E7_49–57_ [102,103,105], and E7_48–54_ [11,106], or E7_86–93_ [102], or oncofetal antigens such as OFA 1, OFA 2, and OFA 3 [107], were used as anticancer antigens.

In addition, next-generation antigens, including personalized peptide antigens (neoantigens), multifunctional/multivalent peptide-based antigens, hybrid peptide-based antigens, and peptide-based cocktail antigens, are appealing in clinical trials presenting promising anticancer potential [9]. Notably, among a specific class of modified antigens, altered peptide ligands, xeno-antigens, analog peptides, or functional mimics of wild-type epitopes (mimotopes) are worthy of mention as considered promising cancer vaccine immunogens [108,109,110,111].

Short peptide-based antigens are attractive due to, inter alia, selective targets in the immune system. On the other hand, it should be highlighted that immunogenicity from peptide antigens is still not enough and the presence of vaccine adjuvants is needed to magnify immune responses during vaccination.

Many imperfect adjuvants, such as aluminum salts, have been widely used so far. Other available better adjuvants, such as oil emulsions, Toll-like receptor (TLR) ligands, or virosomes, still are insufficient due to their inter alia structural heterogeneity [112,113]. In recent years, short peptide-based assemblies as homogenous peptide antigens have been investigated because of the possibility of exhibiting multivalent antigens on the assembled peptide surface.

Peptide OVA-Q11 is the first peptide vaccine adjuvant determined by Collier and coworkers [114]. For this purpose, they linked antigen OVA_323–339_ together with a domain Q11 (QQKFQFQFEQQ), resulting in well-defined nanofibers. The authors proved that this peptide enhanced the immunogenicity.

In addition, self-adjuvanting antigens were developed from nanofibrils with B cell epitopes disposing on their surface via the attachment of the Q11 domain to MUC1-derived epitopes with varied glycosylated threonine. They also attached antigen OVA_323–339_ to the self-assembled peptide KFE8 (FKFEFKFE) [114]. OVA-KFE8 formed nanofibrils and activated a strong antibody response analog to OVA-Q11.

Rudra et al. reported the enantiomeric effect of nanofibrils on the immune response of OVA epitopes by changing the natural D-amino acids to L-amino acids in the Q11 domain [115]. They characterized enantiomeric nanofibrils and found that fibrils consisting of D-analogues increased antibody response and extended antigen presentation, pointing to the developed efficiency of D-peptides in vaccines as well as the stereochemistry-related biological materials in regulating the immune system. It proved the facilitation of vaccination by using peptide assemblies as homogenous vaccine adjuvants.

Yang et al. formed hydrogels from self-assembled peptides to develop adjuvants that can facilitate administration and correct biosafety [116]. They checked the impact of enantiomerism on the forming hydrogels on vaccination via the synthesis of peptide gels containing both D- and L-amino acids. Examples can be Nap-G^D^F^D^F^D^pY-OMe and Nap-GFFpY-OMe, undergoing hydrogelation initiated by alkaline phosphatase (ALP)-induced dephosphorylation. Co-assembly of the hydrogels with OVA supported the hydrogelation, suggesting the powerful uptake of OVA in the gelators. Both the enantiomeric self-assembled adjuvants resulted in potent formation of immune antibodies and cytokines secretion because of the increased cellular capture of antigens, maturation of dendritic cells, antigen accumulation at lymph nodes, and generation of germinal centers. Specifically, the D-peptide hydrogels, contrary to L-analogues, had improved results in OVA accumulation and prevention of tumor growth. Next, researchers slightly synthesized hydrogel adjuvants from enantiomeric gels Nap-G^D^F^D^F^D^Y and Nap-GFFY to simplify adjuvant, preparation avoiding enzymatic hydrolysis [117].

The thixotropy of the resultant gels permitted powerful encapsulation of antigen OVA. Moreover, the gels encapsulated X-ray-weakened tumor cells, playing the role of antigen therapeutic agents to repress tumor growth and increase survival by activating the CD8^+^ T cell pathway. Nap-G^D^F^D^F^D^Y gels showed advanced triggering of the immune response. Numerous peptide gelators extracted from the Nap-GFFY or Nap-G^D^F^D^F^D^Y^D^ were engineered for adjuvants. Tracing Nap-G^D^F^D^F^D^Y^D^ with a positively/negatively charged residue at the C-terminus resulting in Nap-G^D^F^D^F^D^Y^D^K and Nap-G^D^F^D^F^D^Y^D^E can be a good example. In the case of the encapsulation of OVA, the gelators built from the positively charged peptides demonstrated improved inducing immune responses [116]. Drug-modified vaccine adjuvants were developed, otherwise, by the replacement of naphthalene by nonsteroidal anti-inflammatory drugs at the N-terminus of G^D^F^D^F^D^Y [116]. The gelators uptaking OVA demonstrated exceptional capacity for tumor elimination due to combining the anti-inflammatory features of therapeutic agents. Whereas, the incorporation of MUC1 epitopes into Nap-G^D^F^D^F^D^Y^D^K led to corrected immunogenicity of antigen MUC1 [96].

Notably, DP7 (VQWRIRVAVIRK), a new cationic hydrophilic antimicrobial peptide connected to cholesterol (DP7-C), is bifunctional—playing the role of carrier and immunoadjuvant [118]. DP7-C can effectively deliver diverse antigenic peptides (to above 75% of the DCs). This peptide may stimulate both dendritic cell maturation and pro-inflammatory cytokine release on the way of TLR2-MyD88-NF-κB, simultaneously. It is helpful in the improvement of antigen presentation efficiency. In addition, it is an immune adjuvant. DP7-C/neoantigen-pulsed dendritic cell vaccines, based on direct incubation of DP7-C with neoantigens, increased the anticancer efficacy of dendritic cells effectively. It is related to the promotion of the monocyte-derived dendritic cells (MoDC) uptake and presentation in patients with advanced lung cancer. In addition, DP7-C can deliver microRNA engaged in the remodeling of the cancer microenvironment. The cytotoxicity and transfection efficiency are better in comparison with Lipo2000 (lipofectamine 2000) and PEI25K (polyethylenimine 25K) [119]. On the other hand, in the context of DP7, a short dendrimer peptide (KK2DP7) nanoparticle can correct the targeting of lymph nodes (LNs). It prevents cancer recurrence [120] together with immunosuppressants.

Moreover, the peptide delivery platforms will be developed for the delivery of nucleic acid vaccines, particularly mRNA, due to the susceptibility of negatively charged mRNAs to electrostatic binding with cationic peptides [121,122].

**Table 2:** Short peptide-based antigens and adjuvants [9].

Antigens		
OVA_257–264_	SIINFEKL	[91]
OVA_253–266_	EQLESIINFEKLTE	[123]
OVA_323–339_	ISQAVHAA-HAEINEAGR	[93]
OFA 2	ALCNTDSPL	[107]
NY-ESO-1	SLLMWITQV	[99]
MAGE-A3	FLWGPRALV	[101]
Tyrosinase_1–9_	MLLAVLYCL	[83]
Tyrosinase_368–376_	YMDGTMSQV	[83]
MART-1_26–35_	EAAGIGILTV	[84]
gp100_280–288_	YLEPGPVTA	[86]
gp100_209–217_	IMDQVPFSV	[86]
HGP100	KVPRNQDWL	[87]
TRP2	SVYDFFVWL	[89]
Survivin-2B_80–88_	AYACNTSTL	[124]
E-75	KIFGSLAFL	[97]
WT1Pep427	RSDELVRHH-NMHQRNMTKL	[98]
E7_11–20_	YMLDLQPETT	[102]
E7_86–93_	TLGIVCPI	[102]
E7_43–57_	GQAEPDRAHYNIVTF	[103]
E7_49–57_	RAHYNIVTF	[11]
E7_48–54_	PDRAHYNI	[106]
**Adjuvants**		
KFE8	FKFEFKFE	[114]
Q11	QQKFQFQFEQQ	[114]
Hydrogel	Nap-G^D^F^D^F^D^Y^D^	[117]
Hydrogel	Nap-G^D^F^D^F^D^Y^D^K	[125]
Hydrogel	G^D^F^D^F^D^Y	[125]

## 4. Specificity of Short Peptide Assembly in Immunotherapy

In the context of immune stimulation, peptides are successful in (pre)clinical studies. However, they have poor immunogenicity in the clinical phase. The reduction of the protein sequences to partial epitopes impairs the affinity of peptides to targeting receptors as well as improves the enzymatic breakdown of peptides [9].

Mentioned above problems can be overcome by self-assembled peptides in the form of well-defined hierarchical nanostructures such as nanoparticles [126], nanotubes [127], nanoribbons [128], or nanofibers [129], driven via noncovalent interactions such as hydrogen bonding interactions, π-based interactions, and hydrophobic interactions. These nanostructures are biocompatible and biodegradable; hence, they can be the perfect scaffolds for delivering and displaying peptide therapeutic agents [112,130]. This nano-approach corrects the circulation of drugs as well as improves the affinity with targeting receptors and other molecular targets resulting from the multivalent outcome. Moreover, the active or passive targeting facility of nano-peptides makes it easier to accumulate drugs at the site of the tumor. Thus, peptide-based biomaterials have the great potency to be either vaccine adjuvants to correct antigen immune response or delivery systems for immunotherapeutics such as vaccines, antigens, genes, checkpoint blockades, or their combinatorial therapeutic agents with conventional drugs [9].

In recent years, substantial progress in the development of self-assembled peptide-based cancer vaccines that produce tumor-specific CD8^+^ T cells inducing tumor killing has been observed [131].

Collier et al., for example, employed self-assembled peptide-based nanofibers to play the role of immune adjuvants [114]. They engineered a multi-epitope cancer vaccine (P/Tr/Td) via co-aggregation of Coil29 (QARILEADAEILRAYARILEAHAEILRAD) together with the B-cell epitope PEPvIII (LEEKKGNYVVTDH), the CD8^+^ T-cell antigen Trp2 (SVYDFFVWL), and tetanus toxoid CD4^+^ T-cell epitope Td (FNNFTVSFWLRVPKVSASHLE). It resulted in nanofibers via non-covalent interactions [132]. Thus, multiple peptide antigens could be delivered at the same time. Epitope peptides are exhibited on the nanofiber‘s exterior, which forms hydrophilic surfaces for simple recognition. It enables coexistent improvement of B cell, CD8^+^ T cell, and CD4^+^ T-cell responses to unique epitope compositions. It leads to promising anti-melanoma activities [133].

The majority of peptide nanogels have been first checked as vaccine adjuvants in immunotherapy because of their non-immunogenic properties.

Shi and colleagues changed the structure of the MAGE-A1 antigen peptide (SQYGEPRKL) via covalent attachment with TLR2 agonist N–Ac PamCS using diverse linkers. The conjugates self-assemble into nanoparticles related to their hydrophobicity and hydrophilicity features and play the role of dual adjuvants, making it easier to passively antigens targe in dendritic cells [134].

Xing and coworkers engineered injectable supramolecular hydrogels through co-assembling poly-L-lysine (PLL) and FF279 dipeptide by electrostatic interactions. Fmoc-FF/PLL-SH gelators have a nanofibrous form with α-helical conformation. It mimics natural antigens; thus, it can act as an adjuvant. During injection around a tumor, the gels activated T-cell responses and inhibited the growth of the tumor. Other adjuvants or antigens were not needed. Thus, the cancer cells themselves were the antigen source to activate the immune response [135].

Xu and colleagues identified the significant relevance of the configuration and the number of lysine residues in the peptide to the enhancement of CTL response. They synthesized NF-kB-activating nano-adjuvant hydrogel via pH-triggered self-assembly of Ada-GFFYGKKKNH2 peptide. They noted that nano-adjuvants with D-configured peptides and 3 lysine-encapsulated antigens via charge-charge interaction were better than 2 lysine residues; hence, a more robust adaptive and innate immune response was generated [131,136].

### 4.1. Delivery Systems

Poor immunogenicity is an ongoing problem of traditional cancer vaccination. Immunosuppression, low production of T cells, and poor T-cell infiltration are the main reasons [137] for the repetition of vaccination. New delivery systems targeting antigen-presenting cells and helping the production of antigens could correct the immunogenicity and consequently lead to positive clinical efficiency. Peptides, especially peptide-based hydrogels, are attractive delivery platforms due to their essential advantages, including great bioactivity, multifunctionality, high water content, increased drug stability and loading capacity, targeted delivery, and excellent biodegradability and biocompatibility [31]. They can have diverse structural modifications and targeting [122]. In consequence, these good characteristics are correlated with greater drug (and peptide) stability, minimalized degradation rates [138], drainage to lymph nodes, and corrected vaccine immunogenicity [139].

The precisely customizable features of self-assembled peptides, contrary to conventional biomaterials, proffer huge potential as delivery platforms for vaccines. The regulation of the aggregating properties of peptide-based therapeutic agents also permits the setting up of self-delivering systems, called drug amphiphiles [140]. Additionally, self-assembled drug amphiphiles enlarge antigen density on the platform’s surface and remove unwanted ingredients in drug formulation, thereby facilitating drug administration and reducing costs of production. The development of peptide delivery systems for vaccines by the combination of the traditional approach with cutting-edge development strategies is a promise to improve their immunogenicity.

In particular, the antigen amphiphile DiC16-OVA, developed by Tirrel et al. via the connection of two palmitic chains to a cytotoxic T-cell epitope from ovalbumin [such as OVA_253–266_ (SIINFEKL)], was the pioneering antigen amphiphile [123]. Self-assembly of DiC16-OVA amphiphile has the form of a cylindrical micelle (diameter of ~8 nm, length of ~200–500 nm). This micelle contains a hydrophobic core with alkyl tails and a hydrophilic surface with antigen epitopes. The antigen micelle is stable in physiological conditions. It is a good feature from the point of view of carrying an accumulation of antigens to lymph nodes. In vivo studies revealed the suppression of tumor growth and the extension of the time of life by the triggering of cytotoxic T-cell immune responses. Thus, the peptide-based nanostructure of antigen amphiphile is a proper approach to antigen delivery and self-adjuvanting systems of antigens to stimulate cellular immunity [9].

Wang and colleagues reported glutathione-responsive nanoconjugates via co-assembly of a negatively charged protein ovalbumin (OVA) with a positively charged cell-penetrating CWWRCRCRC peptide through electrostatic interactions. It should be added that after internalization via antigen-presenting cells, intracellular glutathione destroyed the disulfide bonds of the peptide, activating the fast release of the antigen into the cytoplasm, and next it was cross-presented to stimulate potent CD8^+^ T-cell response. The peptide nanocomposites corrected antigen uptake via dendritic cells, supported stimulation and maturation of dendritic cells, and increased cellular and humoral immune response [131].

Moreover, peptide-based assemblies were used to deliver multiple antigens with an adjustable dose ratio. Collier et al. reported a method of formation of peptide nanofibrils that can incorporate multiple proteins, which at the same time maintain bioactivity. The proteins were linked to βTail (a sequence MALKVELEKLKSELVVLHSELHKLKSEL) that slowly changed conformation in solution from an α-helix to a β-sheet. Dissolution of the βTail fusion protein (e.g., βTail-GFP) together with peptide Q11 that quickly self-assembles to nanofibers results in effective incorporation of fusion proteins into nanofibers. This strategy was useful in the controllable integration of diverse proteins (such as βTail-GFP and βTail-cutinase) into nanofibers. A regulated multi-antigen vaccine scaffold (including controllable antigenic dominance related to the dose of each antigen and the booster formulation) was engineered by the connection of the adjuvanticity of a single fusion protein able to exact integration of multiple proteins [131].

The multi-antigen vaccine scaffolds have the benefits of either concurrent immunity toward diverse pathogens or high affinity toward a single pathogen. In consequence, it has a huge potency in cancer vaccines [9].

Self-assembled peptides have relevance in the co-delivery of immune cell vaccines with other therapeutic agents.

Self-assembled dipeptide FF is the most popular hydrogelator. It has excellent self-assembly ability. Zhang and others reported Fmoc-F-FF-DOPA gel that can bond Fmoc-F and FF-DOPA upon thermolysin activation. They encapsulated chemokine CXCL10, which may employ activated T cells, in the hydrogel. This conjugate exhibited sustained release over 12 days [119]. Qi and coworkers developed the peptide FEFFFK in the form of a nanofibrous beta-sheet structure with encapsulated doxorubicin [141].

Moreover, they linked chlorambucil (CRB) with the FFF leading to self-assembly via π···π interactions. Conjugated CRB-FFFK-cyclen demonstrated a significantly lower IC50 against cancer cells [76]. Recent advances in numerous short peptide-based conjugated hydrogelators, including their morphological modifications in response to stimuli such as pH, enzymes, or ionic concentration, are discussed by Hua and Shen [142].

Li et al. reported a tumor-penetrating peptide Fmoc-KCRGDK hydrogel [9,133]. They designed a personalized cancer vaccine, namely the FK@IQ-4T1 vaccine (PVAX), through the encapsulation of attenuated tumor cells and checkpoint blockades within peptide hydrogels. A drug (JQ1) served as the inhibitor for bromodomain and extraterminal protein BRD4, which triggered immune tolerance via controlling intratumoral PD-L1 expression. The peptide gel was formed from one sequence Fmoc-KCRGDK (FK) with two Fmoc groups to trigger peptide assembly and stabilize the gels via π···π interactions. RGD fragments support the tumor-targeting delivery of therapeutic agents. A co-loaded fluorescent dye (indocyanine green, ICG) displaying big photothermal conversion effectiveness supported the release of 4T1 and JQ1 related to the morphological transition of peptide assembly, which was induced through the hyperthermia effect upon exposure to laser irradiation. In vivo studies revealed that the PVAX vaccine can protect from postsurgical tumor recurrence and metastasis by causing a memory immune response, suggesting a strong cancer vaccine for postsurgical immunotherapy [9,143,144]. Chen et al. employed biotin-GDFDFDYGRGD nanofibers to co-assemble with dabrafenib and doxorubicin (DOX) for targeted and synergistic therapy of differentiated thyroid carcinoma [145].

Yang et al. developed a supramolecular peptide-based ‘Trojan Horse’ concept for the nuclear transport of dual antitumor therapeutic agents [146]. Guo and others engineered HCPT-FFFK-cyclen hydrogel for the improvement of the nuclear buildup of hydroxy camptothecin (HCPT) and its action blocking towards drug-resistant tumor cells [76,133].

Furthermore, Yang and coworkers developed a vaccine module by the encapsulation of OVA antigen, exogenous DC cells, and anti-PD-1 antibodies into RADA16 peptide hydrogelators [147]. The amphiphilic peptide called RADA16 (Ac-RARADADARARADADA-NH_2_) contains alternating hydrophilic and hydrophobic fragments and is a suitable gel to generate strong peptide-based nanofibrous hydrogels [148]. Encapsulating DC cells in the gelators leads to continuous cell viability of DC cells; thus, the extension of the duration time at the injection site and the simplification of transit to lymph nodes. The connection of antigens and DC cells evokes either an exogenous or innate DC-based immune response and consequently enhances antigen-specific T-cell immunity. New encapsulating anti-PD-1 antibodies in the gels increased the proliferation/infiltration of intratumoral CD8^+^ T cells via counteraction of the down-regulation of MHC I caused by PD-1/PD-L1 association. The significant inhibition of tumor growth was observed in in vivo and in vitro experiments. Exceptional immunogenicity caused via Gel-DC-OVA + anti-PD-1 was related to the infiltration of CD8^+^ T cells into lymph nodes and attenuation of Treg cells inside the tumor [9].

Moreover, Lynn and coworkers used a novel approach to the forming of a vaccine platform (called SNP-7/8a) related to charge-modified peptide-Toll-like receptor (TLR)-7/8a aggregates changed to self-assemble into nanoparticles of the same size. It supported the accurate packing of different types of peptide neoantigens in terms of adjuvant TLR-7/8a in the nanoparticles. Consequently, the nanomolecules increased uptake via antigen-related cells and stimulated CD8 T-cell immunity to ~50% of neoantigens, resulting in corrected clearance of the tumor. This approach demonstrates an elastic and multifaceted technique for the co-delivery of peptide antigens and agonists in nanoparticles. It simplifies the activation of antitumor T-cell immunity [133,149].

To the improvement of immunotherapeutic effects, self-assembled peptides are also developed for delivery of programmed cell death receptor 1/ligand 1 (PD-1/PD-L1) antibodies and related mimetic peptides, cancer antigens or epitope peptides, chimeric antigen receptor T cells, and so on.

Moreover, the studies on multifunctional self-assembled peptides that would be able to modulate the environment of tumors in the context of combined immunotherapy, leading to accurate effectiveness with reduced side effects, are worth noting [133].

Cui et al. engineered an iRGD (internalizing RGD peptide, CRGDRGPDC)-based supramolecular system (DOCA-PLGLAG-iRGD) that can simultaneously load aPD1 antibodies, IL-15 cytokines, and stimulators of interferon gene (STING) agonists. It offers a promising solution to overcome problems with multidrug delivery. The DOCA is the hydrophobic segment, while the iRGD is a tumor-targeting molecule. All parts existed in the peptide self-assembling hydrogel. After matrix metalloproteinase (MMP)-2 cleavage at the tumor location, the released drugs caused modulation of the tumor immune microenvironment (TIME) and increased anticancer activity [9,150].

Selected short peptide-based vaccines and related aspects are summarized in Table 3, while supramolecular short peptide-based (co)delivery systems and (co)assemblies are included in Table 4.

### 4.2. Other Immuno-Based Approaches

The combination of nanovaccine with the immune checkpoint inhibitor aPD-L1 and the tumor phagocytosis checkpoint inhibitor aCD-47 can promote the anti-tumor effect. The connection of the advantages of peptide antigens and self-assembly components to achieve self-delivery of antigens is a promising approach for personalizing immunotherapy based on neoantigens. Moreover, this kind of hydrogel vaccine may result in the activity and long-term stimulation of the antigen in vivo. It may stimulate the B-cell immune response in an effective way as well as induce a better CD8^+^ T anti-cancer immune response [9,157,204].

Law and colleagues developed a self-assembly hydrogel related to KLD-12 peptides (Ac-KLDLKLDLKLDL) and the SGRSANA peptide as a substrate of the urokinase plasminogen activator (uPA). The latter is an extracellular matrix enzyme in terms of modifying and tumor invasion on surrounding issues. The uPA is helpful in the diagnosis of malignant breast, urogenital, or gastrointestinal cancers. In the design process, flanking the uPA substrate via KLD-12 peptides is implicated to self-assemble into the gel encapsulation of the KLA peptide sequence (KLAKLAKKLAKLAK). It induces apoptosis in cancer cells. The gel reaches the tumor surroundings, where uPA action is high. This activity disrupts the hydrogel via action on its substrate; hence, releasing the KLA peptide, which can act on tumor cells [205]. The researchers adjusted the degradation kinetics of formulations and apoptotic potential through the changing correlation of the encapsulated and the self-assembled peptides [205].

Furthermore, self-assembled peptides are investigated along with a platform to keep pyruvate kinase M2 (PKM2) tetrameric form against the Warburg effect in renal cell carcinoma. The KLVFF motif derived from β-amyloid to stabilize serine (the natural PKM2 ligand) was used. This platform can restore tumor cell sensitivity to first-line anticancer therapeutic agents; thus, returning this type of cancer’s specificity concerning chemotherapy resistance [150].

A peptide thioester moiety of NBD-DFDF, containing an aminoethyl thioester, can serve as substrates of thioesterases for immediately targeting the Golgi apparatus of cells. Hydrolyzed thiopeptides accumulated in the Golgi apparatus were additionally enriched in the endoplasmic reticulum. It disarrayed protein trafficking via the stress of the endoplasmic reticulum, resulting in the death of different cancer cells with various IC50 values [206].

In recent years, novel assembly strategies with remarkable medical value in modern disease theranostics have been developed. According to a recent study, peptide-assembled biomaterials may be constructed in situ and with appreciable efficiency when bound to cell receptors. An example is a dispersible peptide G7 (GNNQQNY), inspired by the yeast protein Sup35, presenting a prion-like domain that results in the generation of a persistent aggregate [207]. Wang et al. engineered TRA peptide antiCD3-G7-RGD (AKMGEGGWGANDYGNNQQNY-RGD) for immunotherapy, related to bispecific T-cell engager antibodies. The antiCD3-G7-RGD molecule activated T cells in vitro via aggregation of CD3 receptors, while the RGD increased tumor cell attachment and cytotoxicity [8,207].

A layer-by-layer approach to synthetic gel can be used as a platform to study the development of cancer. In addition, this strategy using short self-assembled peptide hydrogels mimicking the extracellular matrix may be helpful for 3D bioprinting applications. It significantly corrected the material’s mechanical strength without reducing its biodegradability [34].

Photothermal and photodynamic therapies, often supported by immune regulation, are emerging anticancer methods [208]. Yan and others developed a co-assembly of the water-soluble immunoreactive thymus peptide RKDVY-TP5 and the photosensitive indocyanine green via modulation of hydrogen bonds to obtain nanomaterials. It led to the optimization of the photophysicochemical profile of the photosensitive moieties, thereby entering a long-acting immunomodulation. It can be the perfect therapy for simply metastasized stromal-encapsulated pancreatic cancer [20,133].

## 5. Combined Peptide-Based Therapies

### 5.1. Immuno-Chemotherapy

To increase the efficiency of cancer treatment, reverse drug resistance, and limit side effects, chemical immunotherapy is becoming progressively important. Liang and colleagues reported a ‘trident’ molecule Nap-CPT-HCQ-pY, with phosphotyrosine for enzymatic assembly, camptothecin (CPT) for chemotherapy, and hydroxychloroquine (HCQ) for inhibition of autophagy. Phosphoric acid triggered Nap-CPT-HCQ-pY to generate the nanobrush first. Nap-CPTHCQ-pY has undergone a hydrophilic-hydrophobic-hydrophilic modification to the nanobrush-nanoparticle-nanofiber structure. It resulted in the slow release of CPT and HCQ for autophagy inhibition-assisted chemotherapy. Combining chemotherapy and checkpoint-blocking therapy, Nap-CPT-HCQ-Y stimulated systemic anti-cancer immunity and consequently the effective suppression of either primary or distant tumors in breast cancer [9,209].

Cui and coworkers conjugated the anti-cancer drug paclitaxel with iRGD to generate a self-assembling peptide paclitaxel-iRGD, and next, they encapsulated CD47 antibodies to produce a hydrogel (aCD47/PF). Paclitaxel causes hydrophobic action, while iRGD allows more charge-attracting action. In the context of adjunct therapy after surgery, an aCD47/PF gelator may be injected into the surgical site to consistently release paclitaxel and aCD47 to remove potential tumor cells in regions that can have been missed during surgery [150].

The exploration of the incorporation of anticancer drugs into self-assembly is worth noting. More specifically, one of the strategies is to reduce the self-assembled molecule as the delivery system for carrying drugs, thereby increasing their bioavailability and mode of action [207]. As an alternative, inducing self-assembly via peptide functionalization with anticancer drugs resulted in a one-pot process with advantages such as eco-friendly synthesis in water media, highly controlled drug release, or the possible inclusion of pigments for imaging and phototherapy. This approach has promising results but is still in the early stages [210].

Marciano and others significantly improved nanoscale peptide filament stability, including the matrix metalloproteinase-9 (a cytokine and chemokine activator for the remodellation of tissue) divisible site through acetylation of its N-terminal site, resulting in the improvement of the form of their targeted gold-based anticancer agents [211].

### 5.2. Immuno-Radiotherapy

Notably, an appealing strategy in effective and safe anticancer therapies combines immunotherapy with other treatment methods [120].

Radiotherapy may cause immunogenic cell death, resulting in vaccine results and modification of the tumor environment. Nevertheless, radiotherapy needs support due to the restricted presentation of antigen, an immunosuppressive environment, and persistent inflammation in the tumor. Wang et al. utilized EISA together with immunogenic cell death induced by radiotherapy to engineer in situ nanovaccines [133]. The peptide Fbp-G^D^F^D^F^D^pY, catalyzed by alkaline phosphatase, was self-assembled into fibrous nanomolecules around the tumor. This allowed for the grasping and encapsulating of autologous antigens. This in situ nanovaccine significantly corrected either the antigen aggregation in lymph nodes or the ‘cross-presentation of antigen-presenting cells’. In consequence, it remarkably improved the effectiveness of radioimmunotherapy [212].

Another interesting issue is a novel approach to utilizing gas-delivery supramolecular peptide-based therapeutic agents in radiotherapy sensitization. The hypoxic tumor environment is a main source of serious radioresistance in solid tumors, as oxygen is needed to repair the DNA destruction caused by radiation. To overcome this problem, a hydrogel of supramolecular peptide-based therapeutic agent as a nitric oxide depository for constant ‘as required’ delivering nitric oxide was developed. It increased tumor radiosensitization. A nitric oxide prodrug glycosylated diazeniumdiolate was linked to an assembled peptide of Nap-FFGGG, leading to Supra-nitric oxide. The galactose may be eliminated through *β*-galactosidase, leading to an ‘uncaged-nitric oxide’ donor and freeing two molecules of nitric oxide. The duration and dosage of nitric oxide therapy may be regulated to attain radiosensitization. This is presented via its possibility of unmediated reaction with DNA at a prompt release of a big dose; hence, repairing the destruction. Moreover, it may optimize the atypical tumor vessels at an assisted release of a small dose. The described strategy can overcome problematic in vivo delivery of nitric oxide [9,133].

## 6. Peptide-Based Cancer Imaging

Short peptides have a simple structure, do not cause many immune responses, and are chemically stable for modifications. They have small binding domains and diverse surface charges.

Thus, they are better than nucleic acid aptamers. In diagnosis, peptides may be useful as natural substrates in protease-based cancer tests. They serve as attractive materials in biosensing to find cancer indicators [213]. Notably, biosensing technology is a chance for more sensitivity and effectiveness in early cancer detection and screening than standard methods (such as mammography, magnetic resonance imaging, computerized tomography, endoscopy, and colonoscopy) [214].

Wei et al. developed peptide adjoints called Vim-TPE-1 and Vim-TPE-2 utilizing peptide CVNTANST that targets vimentin [215]. It was helpful in the experimental study on fluorescence detection of invasive ovarian cancer with malignancies [8]. Yu and others engineered a dual-targeting fluorescence/MR probe to find and mark the edges of cancer of the kidney cell. They mixed gadolinium(III)-doped carbon molecules with the CLT1 peptide and the AS1411 DNA aptamer. It resulted in a pair of homing assays called AS1411-CLT1-Gd-CDs [216]. Liu and coworkers utilized the Ac-KLVFFAL-NH2 peptide to form PNTs and mixed a water-repellent methylglyoxal probe (DBTPP) and a water-soluble probe (IR783) into the PNTs to form a D/I-PNT nanoprobe. It was used for superior-resolution fluorescence analysis in solid tumors [210]. Yan and colleagues developed a twin-mode tool that can be used for MRI and near-infrared fluorescence scanning. They employed self-assembled CyFF-Gd for the detection and separation of cancer tissue in living organisms [217].

The effectiveness of immune checkpoint blockades is related to the expression levels of immune checkpoint moieties. The dynamic nature of this expression is visible during cancer progression and treatment. Therefore, non-invasive in vivo detection of immune checkpoint expression in a highly sensitive manner is critical. The penetration of tissue and long half-life of radiolabeled immune checkpoint antibodies have restrictions; hence, potentially leading to extended clearance from the body and following unneeded toxicity. Radiolabeled peptides playing the role of diagnostic probes have exceptional advantages such as robust tumor infiltration, imperceptible toxicity, and favorable half-life characteristics. The ^64^Cu-labeled DPPA-1 peptide has remarkable efficacy in the precise visualization of PD-L1 expression in cancer tissues; thus, it can serve as a radiotherapeutic agent [218]. Moreover, ^68^Ga-GP12 (^D^TBP-3), a positron emission tomography (PET) tracer, may target TIGIT in advanced non-small cell lung cancer in a specific way. The imaging results obtained with ^68^Ga-GP12 PET/CT are similar to findings related to the widely used ^18^F-FDG PET/CT but with special benefits in the identification of lymph node metastases [219].

## 7. Peptide-Based 3D Culture

Cell culture is the main strategy for verification of the in vitro efficacy of anticancer therapeutic agents. However, cells grown on 2D platforms are not able to mimic the structural connections of tissues. In addition, cell viability, proliferation, differentiation, and sensitivity to pharmacological stimulation may be impacted. Supramolecular peptide hydrogel systems may carry either diverse drugs or may be employed for the formation of the microenvironment needed for cell growth, ensuring 3D support to some extent [9,220].

Song and coworkers synthesized the RADA16-I peptide with an isosmotic sucrose solution to form a solid 3D cell culture. In hydrogel, integrin beta1 was clearly expressed, which was related to the generation of tight intracellular junctions. Thus, the gel was a preferable microenvironment for the growth of ovarian tumor cells. It should be mentioned that the cells in the RADA16 group were irregularly elongated into the gelator; thereby, this gel was a proper environment for cancer cells to grow. In addition, CDDP and PTX presented remarkably reduced inhibitory rates in RADA16-I cultures compared with 2D cell cultures with the same cell counts. It was favorable to screening chemotherapeutic agents sensitive to tumor models [221]. Nevertheless, the low pH of RADA16-I could lead to the destruction of the encapsulated cells and host tissues. Thus, Huang and others selected the peptide FLIVIGSIIGPGGDGPGGD to combine with a complete culture medium. It was the construction of a hydrogel system useful for the 3D cultivation of breast tumor cells [222].

Nagai and coworkers reported a self-assembled peptide [CH_3_CONH]-RLDLRLALRLDLR-[CONH_2_] capable of keeping stability in a neutral solution and being sterilized by a sterilizer. The researchers noted that the hydrogel culture system presented a well-fitted platform for cell adhesion/survival [223]. Haines-Butterick and others employed the longer peptide VKVKVKVKVKVDPLPTKVEVKVKV to transport C_3_H_10_t1/2 mesenchymal stem cells. The form of gel was destroyed during the syringe injection; however, immediately after the finished action, the gelator rapidly reset and came back to its previous mechanical hardness. It led to the ideal vehicle for the delivery of cells to discrete biological regions throughout tissue regeneration [142].

Selected short peptide-based immunotherapies and other anticancer strategies are listed in Table 5.

## 8. Natural Short Peptides as Anticancer Therapeutics

Among the novel anticancer therapeutic approaches, bioactive peptides derived from native proteins have shown a tendency to play the role of therapeutic agents against tumor cells [229,230]. Glycine, due to its structural role (beta-turns) and its ability to facilitate cyclization and arginine, for its role in cancer therapeutics, are leading in peptides targeting tumor cells [231]. In addition, apart from glycine, cysteine, lysine, isoleucine, and tryptophan were observed in different places of anticancer peptides [22]. Interestingly, magainin (H-Gly-Ile-Gly-Lys-Phe-Leu-His-Ser-Ala-Lys-Lys-Phe-Gly-Lys-Ala-Phe-Val-Gly-Glu-Ile-Met-Asn-Ser-OH), extracted from *Xenopus laevis* was the first anticancer peptide [230].

Bioactive peptides (Table 6) targeting cancer cells bind unspecifically to negatively charged molecules; hence, they are of exceptional interest [232]. These targets are, inter alia, phospholipids secluded in the inner side of the membrane in normal cells, enabling enhanced specificity [233]. Another feature, such as cholesterol and the microvilli presence on the surface of the tumor, results in greater sensitivity to peptides and fosters selective cytotoxicity [234].

Trinidad-Calderón et al. discussed specific anticancer mechanisms of bioactive peptides [235] (Table 6). These peptides mainly rely on the non-specific mechanism of damaging the cell membrane. Nevertheless, up to now, the majority of these peptides have only been investigated in vitro [236]. Moreover, problems of peptides, such as protease degradation by hydrolysis, fast clearance by kidneys and liver, and instability in the body fluids and gastrointestinal tract, have to be overcome by diverse structural modifications [4].

Furthermore, the enormous potential lies in diketopiperazine-based compounds, which we described elsewhere [237]. Here, we can only mention that these simplest cyclopeptides are common in nature and have a wide spectrum of bio-functionalities. The development of modern theranostics based on cyclic dipeptides has increased attention last time. Modified diketopiperazines are appealing peptidomimetic platforms for future innovative smart drug discovery and delivery systems of drugs that have low permeability as well as cutting-edge biocontrol agents useful in anticancer therapies. Furthermore, we cannot forget that diketopiperazine core is present in numerous known drugs, including anticancer agents such as Plinabulin, Ambewelamide, Phenylahistin, Dehy-drophenylahistin, or Verticilin A [237].

**Table 6:** Natural peptides useful in cancer therapy [235].

Peptides Performing Membrane-Damaging Cell Death		
Decoralin	SLLSLIRKLIT	[238]
MP1	ILGTILGLLKSL	[239]
Tachyplesin	KWCFRVCYRGICYRRCR	[240]
Buforin IIb	TRSSRAGLQFPVGRVHRLLRK	[241]
Magainin 2	GIGKFLHSAKKFGKAFVGEIMNS	[242]
**Peptides Performing Apoptotic Cell Death**		
*Cycas revoluta* peptide	AWKLFDDGV	[243]
GG	GPPPQGGRPQG	[235]
LF11	FQWQRNMRKVR	[244]
FK-16 fragment	FKRIVQRIKDFLRNLV	[245]

To sum up, either natural/bioactive peptides or engineered peptides (including chemical-library-derived peptides) can be used as specific theranostics.

## 9. Peptides as Tumor Drugs

Finally, we should emphasize the enormous potential of short peptides as tumor drugs. In many cases, they are easily used to impede protein-protein interaction and the downstream signals activated by this association. Even if many of these peptides are not yet used clinically, their working principles seem efficient. Selective delivery of therapeutics to tumor cells for effective treatment while reducing damage to healthy tissue is one of the key aspects of cancer therapy. In this context, tumor-homing peptides can bind to molecules that are expressed on the tumor cell’s surface, peptides targeting aberrant cellular signaling pathways, and cell-penetrating peptides that can penetrate the cell membrane and are attached to a drug carrier and used for drug delivery cannot be overlooked [19,246,247]. One example can be the work of Di Donato et al., in which they presented that, peptides interfering with the androgen receptor/Filamin A complex may be helpful in the prevention of prostate cancer progression. They showed that an androgen receptor-derived stapled peptide, called Rh-2025u, which has been shown to interfere in the complex assembly in cancer-associated fibroblasts and prostate tumor cells, can overcome the shortcomings of current still unsatisfactory therapies [248]. Giovanelli et al. reported a short peptide, called S1, that mimics the androgen receptor motif related to the interaction of this receptor with the SH3-Src domain and in consequence, reverses the actions in both cell lines. It means that the complex assembly of androgen receptors and Src operates the androgen-induced motor skills. S1 peptide is a promising drug in the fight against breast cancer [249]. Migliaccio et al. pay attention to similar regulation of prostate and breast cancer because of the crucial function of the androgen receptor and ER. The androgen receptor-based polyproline peptide on both cancer cells playing the role of inhibitor is one of the appealing therapies. This dual effect results from the association of these receptors with Src. It is needed for the “proliferative response to hormones or growth factors.” The development of diverse peptides and peptidomimetics possessing perfect stability as well as cell permeability and low immunogenicity is the future direction of anticancer therapy. These moieties target receptor associations with diverse effectors in response to steroids and growth factors. It leads to effectiveness in hormone-responsive and hormone-resistant tumors [250]. Fath and colleagues highlighted that therapeutic peptides promote tumor cell apoptosis by either inhibition or activation of the anti- and pro-apoptotic proteins, respectively. In this context, therapeutic peptides may be attached to cytolytic peptides to enhance the recognition of tumor cells and improve cell penetration. Notably, cytolytic peptides are short peptides with cationic and amphiphilic features, produced mainly in plants and animals, that can penetrate cell membranes and kill cells [247]. Vadevoo et al. list the current anticancer peptide-based medications such as hormone analogs—octreotide, leuprolide, or goserelin. Nevertheless, they mainly emphasize utilizing short peptides as tumor-homing moieties for the delivery of diverse carriers to tumors. Additionally, they discuss tumor-homing peptides that can be conjugated with chemotherapeutics using linkers to the synthesis of peptide-drug conjugates. In addition, the authors mention short peptides assigned to cells that may attach to intracellular proteins and interfere with protein-protein interactions. These options present promising avenues for the future development of tumor-targeted therapies [246].

## 10. Conclusions

This review summarized knowledge on short peptide-based strategies applied in cancer therapies. Benefiting from the specific advantages of short peptides, such as inter alia unique protein-derived structural features, high bioactivity, and extraordinary biocompatibility, the treatment methods under development can revolutionize cancer management.

Short peptides can serve as vaccine (neo)antigens, adjuvants, delivery systems, or immune checkpoint blockades. They show great potential in clinical trials. They modulate immune responses and inhibit tumor growth safely and effectively. Self-assembled peptides have huge relevance in the development of smart (co)delivery systems. The ability of self-assembled peptides to adopt diverse shapes and react to different stimuli in a controllable manner is a stepping stone for the development of cutting-edge anticancer therapies. Nevertheless, due to the complexity of the compounds, precise control over the self-assembly process and clinical translation of self-assembling peptide vaccines is still and long will be a challenge. The interaction of peptide assembly with organelles and pathways of cellular signaling has not been thoroughly explained yet. On the other hand, artificial intelligence and other advanced bioinformatic tools can help to find successful solutions.

In our opinion, modified/conjugated short peptide-based drugs and vaccines are the most perspective directions for future development. It is hoped that this review, uncovering almost unlimited possibilities of short peptides, can attract much more attention to short peptides and stimulate further efforts in research endeavors to address peptide problems and develop error-free immunotherapeutic approaches for cancer treatment and imaging in the future.

## Figures and Tables

**Table 1:** PD-1/PD-L1 (short peptide checkpoint) blockades [9].

Name	Sequence	Ref.
CSBP	ldvflyse	[22]
C25	CVPMTYRAC	[23]
HAC-I	HVIHEGTVVI	[24]
HAC-V	HVVHEGTVVI	[24]
pep-20-D12	awsATWSNYwrh	[25]
^D^PPA-1	NYSKPTDRQYHF	[26]
DTBP-3	GGYTHWHRLNP	[14]
^D^PPA-2	KHAHHTHNLRLP	[26]
DVS3	dpGWSFGKLHWPGS-Pal)	[27]
Peptide-99	Cyclic[FLIVIRDRVFR(Scc)]G	[28]
PDLong1	FMTYWHLLN-AFTVTVPKDL	[29]
TPP-1	SGQYASYHCWC-WRDPGRSGGSK	[30]
AUNP-12	(SNTSESF)2KFRVTQ-LAPKQIKE-NH_2_	[31]
Peptide-57	Cyclic[F(NMe)ANPHLSWSW(NMe)[NLe](NMe)[NLe]R(Scc)]G	[28]
Peptide-71	Cyclic[F(NMe)F(NMe)[NLe](Sar)DV(NMe)FY(Sar)WYL(Scc)]G	[28]
BMS-986189	[(Met)-Tyr-Ala-Asn-Pro-(Dpr)-Leu-(Hyp)-Trp-(Dab)-Trp-(Nle)-(Nle)-Leu-Cys-Gly]	[14]

**Table 3:** Supramolecular short peptide-based vaccines, their delivery systems, and related aspects for cancer therapies [131].

Peptide	Active Ingredient	Application	Ref.
RADA16	Anti-PD-1 + DCs + OVA peptide	delivery system for DC-based vaccine in EG7-OVA tumor model	[147]
K_2_(SL)_6_K_2_	STING agonist	delivery system in MOC2-E6E7 tumor model	[151]
Fmoc-KCRGDK	BRD4 inhibitor + indocyanine green + autologous tumor cells	delivery system for postoperative cancer immunotherapy in 4T1 tumor model	[150]
Ac-I3SLKG-NH_2_	G(IIKK)3I-NH2	delivery system for MMP-2 overexpressing HeLa tumor model	[152]
OVA_253–266_ peptide	OVA_253–266_ peptide conjugated with dialkyl lipid tail and 2 palmitic chains	delivery system plus peptide tumor antigen in EG7-OVA tumor model	[123]
OVA_254–267_-HBc (Hepatitis B core protein)	OVA_254–267_ peptide	delivery system plus tumor antigen plus adjuvant for B16-OVA-Luc tumor model	[153]
Peptide-MHC/ANXA5	Peptide-MHC (pMHC)	antigen for B16-OVA tumor model	[154]
Ada-GFFYGKKK-NH2	OVA peptide	nano-adjuvant for B16-OVA cancer immunotherapy	[136]
Nap-GFFpY-OMe	OVA peptide	vaccine adjuvant for EG7-OVA tumor model	[116]
Q11 (QQKFQFQFEQQ)	Mucin 1 (MUC1) glycopeptide	delivery system plus adjuvant for MCF-7 tumor model	[95]
Ac-AAVVLLLW-COOH	OVA_250–264_ + HPV16 E7_43–57_	TC-1 tumor model	[104]
Cholesterol-aK-Cha- VAaWTLKAa- LEEKKGNYVVTDH	EGFRvIII + PADRE epitopes	cellular and humoral immune response in B16-EGFRvIII tumor model	[155]
Coil29 (QARILEADAEIL RAYARILEAHAEILRAD)	EGFRvIII, PADRE, SIINFEKL, PEPvIII	induction of CD4+ T-cell, and CD8+ T-cell and B-cell responses in mice	[132]
DEAP-DPPA-1	PD-L1 antagonist (DPPA-1) + peptide substrate of MMP-2 + indoleamine-dioxygenase inhibitor (NLG919)	B16-F10 tumor model	[156]
GDFDFDYDX-ss-ERGD (X = E, S or K)		tumor vaccine delivery	[25]
GE11 (EGFR ligand)	Acetylcholinesterase gene + Doxorubicin	drug and gene delivery system targeted toward EGFR-expressing cancer	[157]
S4-8Q (QAEPDRAHYNIVTFCCKCD conjugated to a 4-arm star polymer)	8Q (HPV-16 E7 epitope)	TC-1 tumor model	[11]

**Table 4:** Self-assembled peptide-based diverse types of (co)delivery/(co)assembly in cancer therapy [158,159].

Self-Assembled Peptide	Delivered Molecule	Ref.
Drugs delivery		
AmPDKK_2_/AmPDKK_2_K_4_	doxorubicin	[36]
Ac-ATK(C18)DATGPAK(C18)TA	doxorubicin	[160]
Nap-GFFYGRGDH	doxorubicin	[161]
Fmoc-FK (FK)/Fmoc-FKK (FKK)	doxorubicin	[162]
LLLLLLKKKGRGDS	doxorubicin	[163]
Adenine acetic acid-FFF	doxorubicin	[164]
PEG-QAEAQACA	doxorubicin	[165]
ATKTA-S-S-ATKTA	curcumin	[166]
RGD-PEG-SS-PTX	paclitaxel	[167]
GGVVVRGDR	paclitaxel	[168]
Npx-^D^F^D^F^D^E^D^Y	cisplatin	[25]
chlorambucil-FFFK-cyclen	chlorambucil	[169]
CA-C11-GGGRGDS	methotrexate	[170]
RADA16-I	doxorubicin, curcumin	[171]
Cbz-FF	doxorubicin, curcumin	[172]
EVEALEKKVAALEC KVQALEKKVEALEHGW	doxorubicin, apomorphine, rapamycin, tamoxifen, dexamethasone, paclitaxel	[173]
AAAAAAK	boron neutron	[174]
GRVGPLGK	doxorubicin/paclitaxel/ curcumin	[175]
IDM-GFFYGRGDH	IMD + doxorubicin	[161]
FKFEY-YSV	hydroxycamptothecin + tyroservatide	[176]
Nap-FFYERGD	cisplatin + irinotecan	[177]
HCPT-FFERGD	hydroxycamptothecin + cisplatin	[146]
Rh-GFFYERGD	Rhe + Cisplatin	[178]
Gene delivery		
FF	SiRNA	[179]
RRRR	pDNA	[148]
AAAAAAK	SiRNA	[180]
KKALLHAALAHLL ALAHHLLALLKKA	lentiviral	[181]
Other delivery		
Nap-FFGGG-*β*-Gal	Achieving radiosensitization by the delivery of NO to normalize blood vessels	[182]
DOCA-PLGLAG-iRGD	Delivery of STING agonist and aPD1	[150]
Gene drugs-based co-assembly		
Retro-Inverso CADY-K	siRNA	[183]
KALA-2LMWP-NLS	pDNA	[184]
TR4	pDNA	[148]
Fmoc-RRMEHRMEW	siRNA/AS1411 aptamer	[30,185]
GRVEVLYRGSW GRVRVLYRGSW	siRNA	[186]
STR-H_16_R_8_	siRNA	[187]
STR-HK	siRNA	[188]
TNCP	ASN	[189]
FC-PyTPA	Bcl-2 siRNA + PyTPA	[190]
Chol-HHHHHHH-AKRGARSTA	siRNA + 1-methyl-DL-tryptophan	[9]
Phototherapeutic agents-based co-assembly		
NapFFKYp	indocyanine green	[191]
Z-Histidine-Obzl	biliverdin	[192]
Fmoc-L_3_-OMe	m-5,10,15,20-tetrakis (4-hydroxyphenyl) porphyrin	[9]
H-FF-NH_2_·HCl, Fmoc-K	chlorine6	[193]
Fmoc-L-L	chlorine6	[194]
Fmoc-L_3_-Arg	5,10,15,20-tetrakis (4-hydroxyphenyl) porphyrin	[195]
TAT-IR780	IR780 + doxorubicin	[196]
RKDVY(TP5)	TP5 + indocyanine green	[20]
LND-K	TPPS_4(photosensitizer)_ + lonidamine	[159]
Immunotherapeutic agents-based co-assembly		
10 K-Adpgk	Adpgk (neoantigen peptide)	[197]
Epitope-R_8_	epitope	[198]
AC-KLVFFAL-NH_2_	cyclic diguanylate monophosphate	[120]
Fbp-GDFDFDYD(E, S, or K)-ss-ERGD	OVA (ovalbumin)	[25]
Fbp-GDFDFDYDK(γE)_2_-NH_2_	OVA	[25]
HmA	antibody	[199]
ECPs	K-OVA_257–264_ + E-OVA_323–336_	[200]
DEAP-^D^PPA-1	^D^PPA + NLG919	[156]
PCP	R848 + doxorubicin	[201]
AmpF, AmpFY, AmpFC919	NLG919 (indoleamine 2, 3-dioxygenase (IDO) inhibitor) + ^125^I	[20]
Radiosensitizer-based co-assembly		
Ce6-R9-^125^I-RGD	^125^I + Ce6 + miR-139-5p	[202]
NIA-D1	2-(2-nitroimidazol- 1-yl) acetic acid + R848	[35]
FFRGD	H_2_S + 2-Gy radiation	[203]
HCPT-FFRGD	nuclear delivery of dual anticancer drugs	[146]

**Table 5:** Other supramolecular peptide-based strategies in cancer therapy [133].

NBD-^D^F^D^F-Thioester	Golgi targeting and destruction	[206]
LTP-VEALYL	lysosome targeting and destruction	[207]
Npx-^D^F^D^F^D^K(Pt)^D^pY	in situ nanomedicine and pro-apoptosis	[202]
ABS-GFFKYPLGLAG-PEG1000	conquering radioresistance by inducing CSC differentiation	[150]
^D^N^D^Y^D^S^D^K^D^P^D^T^D^D^D^R^D^Q^D^Y^D^H^D^F	PD-L1 inhibition	[150]
LGASWHRPDKK(PLGYLG-(man)3-)LVFFAECG	bispecific nanoantibody	[150]
Fbp-G^D^F^D^F^D^pY	radioimmunotherapy	[212]
Nap-FFK(CPT)-K(HCQ)-pY	chemoimmunotherapy	[209]
RKDVY-ICG	photothermal immunotherapy	[208]
ligand/receptor	photodynamic therapy	[66]
QRHKPRE (QRH)/epidermal growth factor receptor		[150]
YHWYGYTPQNVI (GE11)		[150]
CMYIEALDKYAC		[150]
WxEAAYQrFL/keratin 1		[224]
LQNAPRS/CD133		[217]
anti-HER2 peptide/human epidermal growth factor receptor 2		[225]
cyclo-[2NaI-Gly-d-Tyr-Arg-Arg] (FC131)/cel-syrface chemokine receptor		[226]
KSD-cha-FskYLWSSK(AE147)/urokinase-type plasminogen activator receptor		[227]
KDKPPR/NRP-1		[203]
EHWSYGLRPG/gonadotropin-releasing hormone receptor		[228]

## Supplementary Materials

The following supporting information can be downloaded at: https://www.mdpi.com/article/10.3390/cancers16193254/s1, Table S1: A snapshot of therapeutic cancer peptide-based vaccine trials and their clinical and immunological outcome.
