# Supplementary material for: Short Peptides as Powerful Arsenal for Smart Fighting Cancer
Source: Cancers (Basel). 2024 Sep 24;16(19):3254. doi: 10.3390/cancers16193254 (PMC11476321; doi:10.3390/cancers16193254)
Supplement: Supplementary file 1 [file cancers-16-03254-s001.zip › cancers-3189145-supplementary.pdf]

Short Peptides as Powerful Arsenal for Smart Fighting Cancer

Joanna Bojarska * and Wojciech M. Wolf

SUPPLEMENTARY MATERIAL

Table S1. A snapshot of therapeutic cancer peptide-based vaccine trials and their clinical and immunological outcome [Makker,2024]

Vaccine name	Cancert type	Trial phase	Ref.
Vaccination with 4 **HLAA**24-restricted peptides	Non-small cell lung cancer	I	[Abdullah,2020]
START study: tecemotide, peptide vaccine	Non-small cell lung cancer	III	[Abdullah,2020]
Multiple peptide (GRN-1201) +chekcpoint inh. Pembrolizumab (NCT03417882)		II	[Abdullah,2020]
Telomerase-derived helper peptides (UCPVax) +chekpoint inh. Nivolumab (NCT04263051)		II	[Abdullah,2020]
NCT05269381, NCT02818426, NCT03715985	lung	I	[Sheikhlary,2024]
NCT02818426, NCT04263051=telomerase-derived helper pept. (UCPVax)+immune checkpoint inhibitor nivolumab , NCT03715985		II	[Sheikhlary,2024]
NCT02654587, NCT04998474		III	[Sheikhlary,2024]
Neoantigen Peptides NCT04487093 Personalised Peptide Vaccine NCT04397926			[Stephens,2021
Tumour-pulsed monocytederived DC vaccine with expanded T cell populations	Ovarian cancer	I	Abdullah,2020]
Personalized neoantigen (NeoVax) + poly-ICLC +immune checkpoint inh. Nivolumab (NCT04024878)		I	[Abdullah,2020]
OSE2101 + Montanide +immune chekpoint inh. Pembrolizumab (NCT04713514)		II	[Abdullah,2020]
NCT05269381, NCT04580771, NCT03728881, NCT02865135, NCT03311334, NCT03761914, NCT02785250, NCT03206047	Cervical/Uterus/Ovarian Cancers	I	[Sheikhlary,2024]
NCT03728881, NCT04445064, NCT03946358, NCT02865135, NCT03311334, NCT03029403, NCT03761914, NCT02785250, NCT03206047, NCT04713514=OSE2101+montanide+immune checkpoint inh. pembrolizumab		II	[Sheikhlary,2024]
NCT04782895, NCT04508309		III	[Sheikhlary,2024]
DCs pulsed with HER2 peptides, administered with anti-HER2 antibody	breast	I/II	[Abdullah,2020]
ESR1 (NCT04270149)+ immunol. Adj. Montanide ISA and GM-CSF		I	[Abdullah,2020]
E75 peptide + GM-CSF		Two-	[Peoples,2005]

		Stage Safety Trial	
HER-2 (NCT04144023)+immunol. Adj. GM-CSF		I	
AE37 peptide vaccine+immune checkpoint inhibitor Pembrolizumab (NCT04024800)		III	
Multipeptide cancer vaccine (PVX-410)+immune checkpoint inhibitor Pembrolizumab+chemotherapy (N CT04634747)		II	[Abdullah,2020]
NCT05269381, NCT02938442),		I	[Sheikhlary,2024]
NCT02938442, NCT03012100, NCT03606967 (neoantgen) NCT02636582, NCT04197687, NCT03606967)		II	[Sheikhlary,2024]
HLA-A* 24-restricted tumour antigen epitope as a peptide vaccine	Esophaheal cancer	III	
Gp100 peptide vaccine with ipiliumab	Metastatic melanoma	III	
NCT05269381	melanoma	I	[Sheikhlary,2024]
*Personalized neoantigen peptides (NeoVax) +immune checkpoint inh. Nivolumab, CDX-301 (NCT04930783) * Personalized neoantigen vaccine (NeoVax) + poly-ICLC + Montanide+immune checkpoint inh. Nivolumab, ipilimumab (NCT03929029)		I	[Abdullah,2020]
Neoantigen peptides + rhGMCSF + anti-PD-1+imiquimod +immune chekpoint inh. Toripalimab (anti-PD-1) (NCT04072900) person. Vac.		I	[Abdullah,2020]
UV1 vaccine + GM-CSF +immune checkpoint inh. Pembrolizumab (NCT03538314)		I	[Abdullah,2020]
UV1 vaccine +immune checkpoint inh. Nivolumab, ipilimumab (NCT04382664)		II	[Abdullah,2020]
Mutated neoantigen peptide (BRAF/CD4 epitopes)+immun. Adj. CD40 antibody and polyICLC (NCT04364230)		II	[Abdullah,2020]
NY-ESO-1 cancer-testis antigen+ Encapsulated in PLGA nanoparticle (NCT0475186)		I	[Abdullah,2020]
Arginase-1 peptide + immunol. Adj. Montanide ISA-51 (NCT03689192)		I	[Abdullah,2020]
NCT03715985=personalized pept. vac. +immunological adjuvant CAF09b		II	[Sheikhlary,2024]
PD-L1 +immuno. Adj. Montanide ISA-51 (NCT03850522)	myeloma	II	[Abdullah,2020]
Rindopepimut peptide vaccine	glioblastoma	III	[Weller,2017]
NCT03149003		**III**	[Sheikhlary,2024]
NCT05283109, NCT05283109, NCT04280848=telomerase-derived helper pept. (UCPVax) + immunol. Adj. Montanide IS-51 NCT04116658=novel multipeptide (EO2401)+immune checkpoint inhibitor nivolumab/bevacizumab , NCT04943718, NCT02960230	**g** lioblastoma/Glioma	**I**	[Sheikhlary,2024]
IDH1R132H peptide+immune checkpoint inh. Avelumab (NCT03893903)		**I**	[Abdullah,2020]
Multipeptide and the immune		**I**	[Abdullah,2020]

modulator XS15 (NCT04842513)+immunol. Adj. Montanide ISA-51			
Telomerase-derived helper peptides (UCPVax) (NCT04280848)+ immunol. Adj. Montanide ISA-51		**II**	[Abdullah,2020]
NCT04280848,		**II**	[Sheikhlary,2024]
NCT04116658= Novel multipeptide (EO2401)+immune checkpoint inh. Nivolumab, nivolumab/bevacizumab , NCT03018288, NCT02960230			[Abdullah,2020]
Neoantigen Peptides (NCT04749641, NCT02358187, NCT01130077)			[Stephens,2021]
Neoantigen Combination Therapy (NCT03893903, NCT02960230)			
NCT05010200=PGV001 (multiple pept.)+immunol. Adj. CDX-301	**prostate**	**I**	[Sheikhlary,2024]
NCT03579654, NCT04114825=RV001V+montanide ISA-51		**II**	[Sheikhlary,2024]
Bcl-xL + immunol. Adj. CAF09b (NCT0 3412786)		**I**	[Abdullah,2020]
RV001V +immun. Adj. Montanide ISA-51 (NCT 04114825)		**II**	[Abdullah,2020]
Novel Peptide-Conjugate NCT04701021			[Stephens,2021]
TARP Peptide NCT02362464			
Telomerase-derived Peptides NCT01784913			
, NCT03761914), NCT05025488, NCT04688385=person. Pept. vac.+immunol. Adj. TLR1/2 ligand XS15	**Leukemia/blood**	**I**	[Sheikhlary,2024]
NCT03559413=person. Pept. vac.+immun. Adj. GM-SCF and imiquimod (neoantigens)		**II**	[Abdullah,2020]
NCT04747002, NCT03560752, NCT03559413=person. Pept. vacc. +immunol. Adj. GM-CSF and imiquimod		**II**	[Sheikhlary,2024]
NCT03761914, NCT04060277, NCT03702231, NCT02802943= Personalised Peptide Vaccine Combination Therapy			
PD-L1 and PD-L2 peptides+immunol. Adj. Montanide ISA-51 (NCT03939234)		**II**	[Abdullah,2020]
Personalised Peptide Vaccine Combination Therapy NCT03361852	**lymphoma**		[Stephens,2021]
NCT02865135, NCT03821272, NCT05269381	**Head/neck**	**I**	[Sheikhlary,2024]
NCT03946358, NCT04369937, NCT02865135, NCT03821272, NCT04445064		**II**	[Sheikhlary,2024]
NCT05269381	**gastric**	**I**	[Sheikhlary,2024]
Multiple peptide (OTSGC-A24)+immune checkpoint inh. Nivolumab, ipilimumab (NCT03784040)		**I**	[Abdullah,2020]
KRAS +immun. Adj. polyICLC (NCT05013216)	**Pancreatic cancer**	**I**	[Abdullah,2020]
Neoantigen peptide Poly-ICLC (NCT 03956056)		**I**	[Abdullah,2020]
Personalized peptide vaccine (PEP-DC vaccine) + immune chekpoint inh. Nivolumab, gemcitabine, capecitabine (NCT04627246)		**I**	[Abdullah,2020]
Neoantigen Peptides NCT03956056			[Stephens,2021]
Personalised Peptide Vaccine NCT03558945			
NCT05843448, NCT03715985, NCT05843448	**bladder**	**I**	[Sheikhlary,2024]
NCT03715985		**II**	[Sheikhlary,2024]

Personalised Peptide Vaccine (NCT03359239)			Sheikhlary,2024
NCT05059821	**liver**	**I**	[Sheikhlary,2024]
DNAJB1-PRKACA peptide+immune checkpoint inh. Nivolumab, ipilimumab (NCT04248569)		**I**	[Abdullah,2020]
NCT04206254		**II**	[Sheikhlary,2024]
NCT04206254		**III**	[Sheikhlary,2024]
Personalised Peptide Vaccine Combination Therapy NCT02950766	**kidney**		[Stephens,2021]
NCT03761914	**colorectal**	**I/II**	[Sheikhlary,2024]
Multiple peptide PolyPEPI1018 Vaccine (NCT03391232)+immunol. Adj. montanide		**II**	[Abdullah,2020]
HER2/neu Peptides (NCT02795988)	**adenocarcinoma**		[Stephens,2021]
Personalised Peptide Vaccine (NCT04627246, NCT02600949)			[Stephens,2021]
KRAS + poly-ICLC +immune checkpoint inh. Nivolumab, ipilimumab (NCT04117087)	Multiple cancers and solid tumors	**I**	[Abdullah,2020]
Personalized peptide vaccine (PANDAVAC) + poly-ICLC +immune chekpoint inh. Pembrolizumab (NCT04266730)		**I**	[Abdullah,2020]
GRT-C903 and GRT-R904 peptide +immune checkpoint inh. Nivolumab, ipilimumab (NCT03953235)		**I/II**	[Abdullah,2020]
Personalized adjuvanted vaccine GEN-009 (synthetic long peptides) +immune checkpoint inh. Nivolumab, pembrolizumab (NCT03633110)		**I/II**	[Abdullah,2020]
IDO and PD-L1 peptides (IO102-IO103)+immune chekcpoint inh. Pembrolizumab (NCT05077709)		**II**	[Abdullah,2020]
Telomerase-derived helper peptides (UCPVax) +immune chekpoint inh. Atezolizumab (NCT03946358)		**II**	[Abdullah,2020]
Personalised Peptide Vaccine NCT03715985			[Stephens,2021]
Personalised Peptide Vaccine Combination Therapy NCT03633110, NCT04266730			
NCT03715985 (neoantigen)	Malignant Melanoma, Non-Small Cell Lung Cancer Metastatic, Bladder Urothelial Carcinoma, Metastatic	**I/II**	[Liu,2022]

References

***** Abdullah, T.; Bhatt, K.; Eggermont, L.J.; O'Hare, N.; Memic, A. Bencherif, S.A. Supramolecular Self-Assembled Peptide-Based Vaccines. *Front. Chem.* **2020**, *8*, 598160.

*Liu, J.; Li, M.; Dang, Y.; Lou, H.; Xu, Z.; Zhang, W. Biosensors and Bioelectronics NIR-I Fluorescence Imaging Tumorous Methylglyoxal by an Activatable Nanoprobe Based on Peptide Nanotubes by FRET Process. *Biosens. Bioelectron.* **2022**, *204*, 114068

*Makker, S.; Galley, C.; Benet, C.L. Cancer vaccines: from an immunology perspective. *Immunother. Adv.* **2024**, *4*, 1-12.

* Peoples, G.E.; Holmes, J.P.; Hueman, M.T., *et al.* Combined clinical trial results of a HER2/neu (E75) vaccine for the prevention of recurrence in high-risk breast cancer patients: U.S. military cancer institute clinical trials group study I-01 and I-02. Clinical *Cancer Res*., **2008**, *14*, 797–803.

* Sheikhlary, S.; Lopez, D.H.; Moghimi, S.; Sun, B. Recent Findings on Therapeutic Cancer Vaccines: An Updated Review. *Biomolecules* **2024**, *14*, 503.

* Stephens, J.A; Burgess-Brown, N.A.; Jiang, S. Beyond just peptide antigens: the complex world of peptide-based cancer vaccines, *Frontiers in Immunology*, **2021**, *12*, 696791.
